# Synovial Cytokines Significantly Correlate with Osteoarthritis-Related Knee Pain and Disability: Inflammatory Mediators of Potential Clinical Relevance

**DOI:** 10.3390/jcm8091343

**Published:** 2019-08-29

**Authors:** Timo A. Nees, Nils Rosshirt, Jiji A. Zhang, Tobias Reiner, Reza Sorbi, Elena Tripel, Tilman Walker, Marcus Schiltenwolf, Sébastien Hagmann, Babak Moradi

**Affiliations:** Clinic for Orthopedics and Trauma Surgery, Center for Orthopedics, Trauma Surgery and Spinal Cord Injury, Heidelberg University Hospital, 69118 Heidelberg, Germany

**Keywords:** osteoarthritis, pain, cytokines, inflammation, synovial fluid

## Abstract

The aim of this study was to identify inflammatory mediators of potential clinical relevance in synovial fluid (SF) samples of patients with knee osteoarthritis (OA). Therefore, radiographic OA severity, knee pain and function of 34 OA patients undergoing unicompartmental (UC) and bicompartmental (BC) knee arthroplasty were assessed prior to surgery and SF samples were analyzed for a broad variety of inflammatory mediators, including interleukins (ILs), interferons (IFNs), C-X-C motif ligand chemokines (CXCLs), and growth factors (nerve growth factor; NGF, vascular endothelial growth factor; VEGF, and stem cell growth factor β; SCGF-β) using multiplex assay. Significant differences were observed between the SF levels of different inflammatory markers. When compared to UC OA, significantly higher concentrations of IL-7, IL-8, IL-10, IL-12, IL-13, IFN-γ, VEGF and CXCL1 were detected in BC OA. Correlation analyses revealed significant associations between OA severity and IL-6, IL-8, IFN-γ, SCGF-β, VEGF, CXCL1. Interestingly, increases in both anti- (IL-10, IL-13) and pro-inflammatory (IL-7, IL-12, IFN-γ) cytokines, as well as growth factors (SCGF-β, VEGF), correlated significantly with the level of knee pain. Poorer knee function was associated with higher IL-6, IL-10, IL-12, IL-13, IL-18, βNGF, SCGF-β, VEGF and CXCL9 levels. In conclusion, this study provides an extensive profile of synovial inflammatory mediators in knee OA and identifies cytokines of potential clinical relevance. In fact, five of the mediators examined (IL-10, IL-12, IL-13, SCGF-β, VEGF) significantly correlate with both knee pain and function.

## 1. Introduction

Osteoarthritis (OA) is one of the major causes of chronic pain. Worldwide ≥ 250 million people suffer from clinically relevant OA [1]. Although a large number of OA patients present with signs of inflammation, such as joint swelling and effusion, OA has long been interpreted as a non-inflammatory “wear and tear” disease leading to loss of articular cartilage. Recent findings provide convincing evidence that inflammatory mechanisms play a pivotal role in the pathophysiology of OA. In both animal models and in humans, OA disease progression is associated with synovial inflammation [2,3]. In OA joints mononuclear cells (e.g., T-cells and macrophages) infiltrate the synovial membrane (SM) and levels of pro-inflammatory mediators in peripheral blood and synovial fluid (SF) samples are elevated [4,5,6,7]. Increased release of inflammatory cytokines and chemokines induces the expression of proteolytic enzymes such as matrix metalloproteinases resulting in cartilage breakdown [8]. Thus, low-grade joint inflammation is considered a key mediator of OA pathogenesis. Recently, we demonstrated that CD14^+^ macrophages are the predominant cell population in SM samples of unicompartmental (UC) OA. In contrast, bicompartmental (BC) OA seems to be driven by both CD14^+^ macrophages and CD4^+^ T cells and shows a higher inflammatory profile with significantly increased concentrations of proinflammatory cytokines in SF samples (e.g., CXCL1, eotaxin, interferon (IFN)-γ, interleukin (IL)-7, IL-8, IL-9, IL-12) [5]. Yet, the clinical relevance of specific inflammatory mediators and cell types remains elusive. Besides triggering degradation of articular cartilage, inflammatory mediators seem to be involved in the development of OA-related pain via interactions between the immune and nervous system. IL-6 and tumor necrosis factor α (TNFα) lead to peripheral sensitization of joint nociceptors in experimental models of OA, resulting in mechanical hyperalgesia [9,10]. Furthermore, OA-induced expression of the chemo-attractant molecule C-C motif ligand 2 (CCL2) can directly activate nociceptive neurons by binding to neuronal C-C chemokine receptors type 2 (CCR2) contributing to pain. Knock-out of the CCL2/CCR2 axis significantly delayed the onset of pain-related behavior in mouse models of OA [11]. Moreover, OA-related joint inflammation modulates the expression of growth factors including nerve (NGF) and vascular endothelial growth factor (VEGF). NGF can increase the expression of TRPV1 (transient receptor potential cation channel subfamily V member 1) and sodium channels on primary sensory afferents and induce the release of Substance P and Calcitonin gene-related peptide (CGRP) [12,13], which is associated with increased OA-related pain [14]. VEGF expression in chondrocytes was reported to be a particular feature of OA when compared to rheumatoid arthritis (RA) [13] and pharmacological blockade of VEGF-receptor 1 resulted in reduced pain-related behavior in experimental OA models [15].

Despite the convincing evidence that inflammatory mediators play a crucial role in OA pathogenesis and the growing body of literature demonstrating a link between inflammation and pain in experimental models of OA, only few clinical studies assessed interactions between synovial inflammatory mediators and OA-related pain and function [16,17,18,19]. Yet, including clinical data in the interpretation of cellular and molecular analyses of inflammatory markers is essential to reveal the mechanisms underlying the complex pathophysiology of OA pain. Thus, the aim of the current study was: (i) to map the cytokine profile of knee OA; and (ii) assess associations between cytokine concentrations and clinical parameters including OA severity, pain and function.

To the best of our knowledge this is the first study analyzing this broad variety of synovial cytokines in correlation to clinical data of patients suffering from knee OA. In brief, we identified five cytokines that significantly correlate with OA-induced knee pain and disability and might become targets of pharmaceutical approaches to treat the excruciating symptoms of knee OA.

## 2. Experimental Section

### 2.1. Study Population

A total of 34 patients with primary knee OA (20 women, 14 men) consecutively undergoing knee replacement surgery at Heidelberg University Hospital were enrolled into this study. OA was defined according to the American College of Rheumatology criteria. Based on anteroposterior, sagittal and varus-valgus stress radiographs, OA was classified as medial UC OA or BC OA. The Kellgren and Lawrence (K&L) scoring system was used to assess the radiographic severity of OA [19]. Patients with UC OA were scheduled for UC and those with BC OA for total knee arthroplasty. Underlying inflammatory diseases including RA, clinical and laboratory signs of systemic inflammation, intake of disease-modifying anti-rheumatic drugs (DMARD) and intra-articular injections of corticosteroids or hyaluronic acid as well as arthroscopy on the target knee within 3 months before enrollment were considered exclusion criteria. The study was conducted in accordance with the local ethics committee of the Medical Faculty at Heidelberg University and the Declaration of Helsinki and was approved by the institutional review board (S333/2007). All patients provided written informed consent prior to study enrollment.

### 2.2. Clinical Assessment

To assess the radiographic severity of OA anteroposterior radiographs of the symptomatic knees were used and graded according to the K&L scoring system (0–4) by the same experienced orthopedic surgeon [19]. Knee pain and function prior to surgery was assessed using the 11-point (0–10) numerical rating scale (0 = no pain; 10 = worst pain) and the 12-item self-administered Oxford Knee Score (OKS-12) [20], respectively.

### 2.3. Sample Collection and Multiplex Cytokine Analysis

SF samples were collected at the time of surgery. Prior to arthrotomy, needle aspiration was performed to remove SF. The samples were stored in sterile tubes at −80 °C until further processing. The length of time between sample collection and cryopreservation ranged from 1 to 3 h. To analyze the cytokine profile of SF samples the Pro-Human Cytokine Multiplex Assay (Bio-Rad, Munich, Germany) was used according to manufacturers’ instructions. The following inflammatory mediators were examined using the Luminex 200 system: IL-1α, IL-1β, IL-2, IL-4, IL-5, IL-6, IL-7, IL-8, IL-9, IL-10, IL-12, IL-13, IL-15, IL-16, IL-17, IL-18, βNGF, Interferon (IFN)-γ, IFN-α2, leukemia inhibitory factor (LIF), macrophage colony stimulating factor (M-CSF), macrophage inflammatory protein (MIP-1β;CCL4), CCL2 (MCP-1), CCL5 (RANTES), CCL7 (MCP3), CCL27 (CTACK), stem cell factor (SCF), stem cell growth factor β (SCGF-β), TNFα, VEGF, C-X-C motif ligand 1 (CXCL1), CXCL9, CXCL12. Bio-Plex Manager version 5.0 (Bio-Rad, Munich, Germany) was used for data processing. Cytokine and chemokine concentrations were calculated by reference to the standard curve. The sensitivity of the multiplex kit was < 5 pg/mL.

### 2.4. Statistical Analyses

Descriptive statistics of demographic and clinical parameters, as well as SF concentrations of inflammatory markers, are expressed as mean ± standard deviation (SD) and range. Gaussian distribution of cytokines was assessed using the D’Agostino & Pearson omnibus normality test. Unpaired Student’s *t*-test was used to analyze differences in cytokine levels between UC and BC OA, showing Gaussian distribution. For cytokines that did not show Gaussian distribution, the Mann–Whitney *U* test was performed to examine differences between UC and BC OA. Due to the predominantly non-parametric distribution of cytokines Kruskal-Wallis test followed by Dunn’s multiple comparison test was used to detect differences between cytokine concentrations in SF samples of the total study population. Spearman’s rank correlation coefficient was used to examine correlations between inflammatory mediators and K&L score, numerical rating scale (NRS) and OKS-12. All reported *p*-values are two-tailed and a *p*-value < 0.05 was considered statistically significant. Statistical analysis was performed using Prism version 6.01 software (GraphPad Software Inc., La Jolla, CA, USA).

## 3. Results

### 3.1. Description of the Study Population

Demographic and clinical parameters of the study population are presented in Table 1.

**Figure 1:**
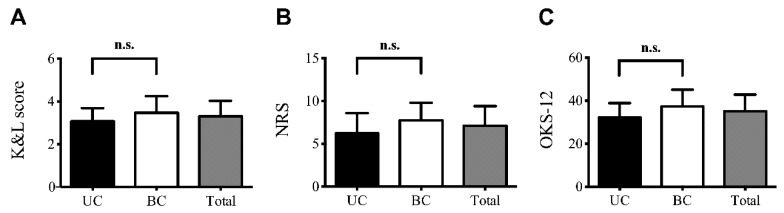
Clinical parameters of the study population. Mean (± SD) K&L, NRS and OKS-12 scores of the total study population (Total) and the patients diagnosed with UC or BC OA are presented. Comparing UC OA with BC OA no significant differences in (**A**) the severity of OA (K&L score), (**B**) knee pain (NRS) and (**C**) function (OKS-12) were observed. Unpaired student’s *t*-test was used for statistical analysis. Data are expressed as mean ± SD.

In brief, a total of 34 patients (58.8% female, 41.2% male) participated in this study. 14 patients were diagnosed with UC OA, whereas 20 patients suffered from BC OA. Mean age (± SD) and body mass index (BMI, ± SD) was 67.38 (± 10.48) years and 30.74 (± 5.78) kg/m², respectively. No significant differences in age or BMI were observed between UC and BC OA. K&L scores ranged from II-IV. The majority of UC OA patients (64.3%) had a K&L score of 3. In contrast, BC OA was graded K&L 4 in 63.2% of the patients. Mean knee pain was rated 7.12 (± 2.29) and OKS-12 score 35.15 (± 7.68). There were no statistically significant differences in K&L, NRS and OKS-12 scores between UC and BC OA as revealed by Student’s *t*-test (Figure 1A–C).

### 3.2. Profile of Inflammatory Mediators in OA-Related Knee Pain

SF cytokine levels and the results of the correlation analyses between inflammatory mediators and clinical parameters are summarized in Table 2.

**Figure 2:**
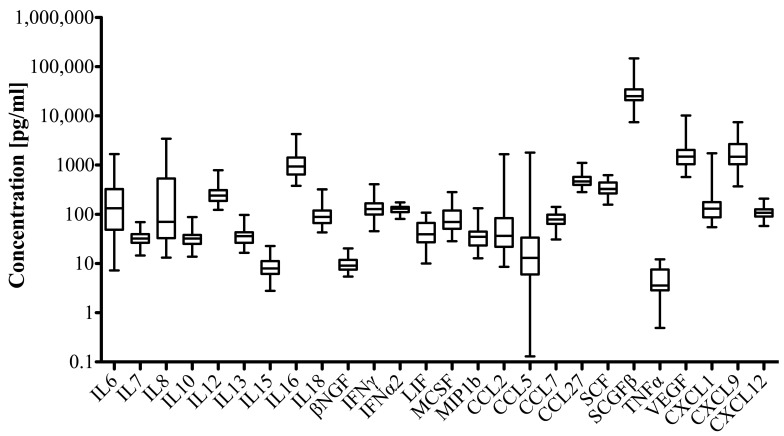
Cytokine pattern in synovial fluid (SF) of patients with knee osteoarthritis. The Pro-Human Cytokine Multiplex Assay (Bio-Rad) was used to analyze the cytokines in SF samples. Cytokine and chemokine concentrations were calculated by reference to the standard curve. The sensitivity of the multiplex kit was < 5 pg/mL. Data are presented as box and whisker plot showing median (interquartile range; IQR) values (box) and minimum to maximal values (whiskers).

Mean (± SD) SF concentrations ranged from 5 (± 3.1) pg/mL for TNFα to 32,337.5 (± 25,341.6) pg/mL for SCGF-β. Growth factors including SCGF-β and VEGF reached highest SF concentration levels followed by CXL9 and IL-16. The overall cytokine profile is presented in Figure 2.

Multiplex analysis demonstrated highly significant differences in concentration levels of different inflammatory markers in SF samples from knee OA patients (Kruskal-Wallis test, **** *p* < 0.0001). No differences were observed between the mean concentration levels of anti-inflammatory interleukins (IL-10, IL-13) and the pro-inflammatory cytokine IL-6. Comparing UC and BC OA, significantly different cytokine levels were seen for the following mediators: IL-7, IL-8, IL-10, IL-12, IL-13, IFN-γ, VEGF, CXCL1 (Table 3). All mediators were higher in SF samples of BC OA patients and 6 of them show pro-inflammatory characteristics (IL-7, IL-8, IL-12, IFN-γ, VEGF, CXCL1).

### 3.3. Correlation of Inflammatory Mediators and Clinical Parameters

SF concentration levels of the different inflammatory mediators were correlated with the severity of OA (K&L scores), knee pain (NRS) and function (OKS-12). Data summary is presented in Table 2. In brief, Spearman’s correlation analysis revealed significant correlations (weak-moderate) between OA severity and IL-6, IL-8, IFN-γ, SCGF-β, VEGF as well as CXCL1. Best associations between K&L scores and inflammatory mediator levels were observed for IL-8 (r = 0.4723, *p* = 0.0055 **) and CXCL1 (r = 0.4931, *p* = 0.0035 **).

Interestingly, both anti- (IL-10, IL-13) and pro-inflammatory (IL-7, IL-12, IFN-γ) cytokines as well as growth factors (SCGF-β, VEGF) correlated significantly with the level of knee pain. Moderate correlations (** *p* < 0.01) were detected for IL-10, IL-12, IL-13 and VEGF. IL-6 and TNFα was not associated with NRS scores.

**Figure 3:**
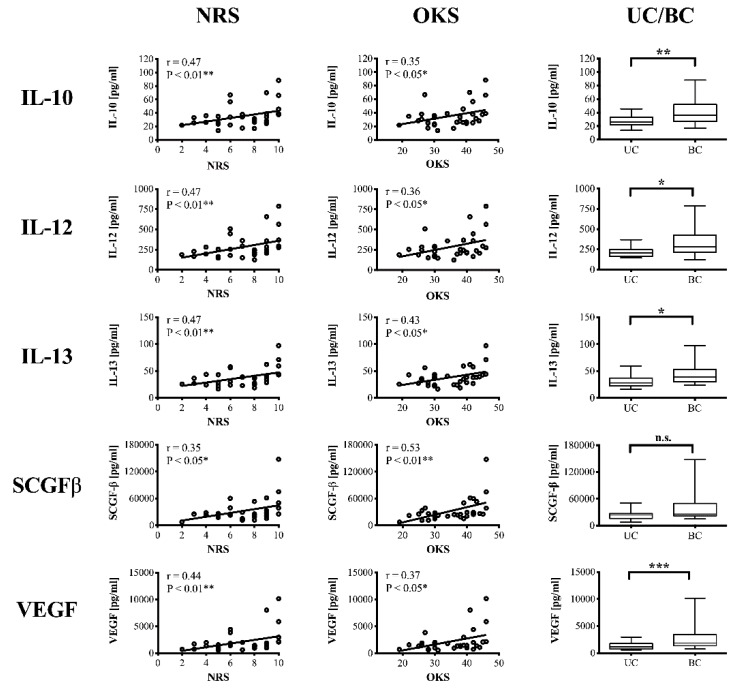
Significant correlations between inflammatory mediators and clinical parameters (NRS, OKS). Correlation analyses were performed using Spearman’s rank correlation coefficient (r). Since IL-10, IL-12, IL-13, SCGFβ and VEGF did not show Gaussian distribution Mann–Whitney *U* test was performed to calculate differences in SF cytokine concentrations between unicompartmental (UC) and bicompartmental (BC) osteoarthritis. *p*-Values < 0.05 were considered statistically significant and are indicated with asterisks: * *p* < 0.05; ** *p* < 0.01. SF = synovial fluid; NRS = numerical rating scale; OKS = Oxford Knee Score; IL = interleukin; SCGF = stem cell growth factor; VEGF = vascular endothelial growth factor.

Correlating inflammatory mediators with knee function (OKS-12) revealed significant relationships for the following cytokines: IL-6, IL-10, IL-12, IL-13, IL-18, βNGF, SCGF-β, VEGF, CXCL9. In summary, 5 of the examined inflammatory mediators (IL-10, IL-12, IL-13, SCGF-β, VEGF) significantly correlated with both NRS and OKS-12 scores suggesting a clinically relevant role in the pathophysiology of knee OA (Figure 3). The growth factors SCGF-β and VEGF additionally correlated with OA severity.

## 4. Discussion

This study provides an extensive profile of synovial inflammatory mediators in knee OA and identifies cytokines of potential clinical relevance. The OA-induced inflammatory pattern is characterized by a wide array of both anti- and pro-inflammatory cytokines as well as growth factors. Studies have extensively assessed cellular and molecular interactions between joint inflammation and cartilage breakdown in the pathophysiology of OA. Among others pro-inflammatory mediators including IL-1β, TNFα, IL-6, IL-15, IL-17, and IL-18 are known to disrupt metabolic homeostasis by promoting catabolic processes and enzymatic cartilage degradation [21,22]. In contrast, only few studies have addressed the link between articular inflammatory mechanisms and pain in patients suffering from knee OA. Yet, the major clinical problem of the patients is disabling pain.

Experimental models of pain demonstrated direct and indirect pro-nociceptive effects of cytokines [23]. On the one hand cytokines can directly activate neuronal cytokine receptors expressed on proportions of sensory neurons including joint nociceptors and initiate pain transmission. On the other handy cytokines induce the release of other neuroactive inflammatory mediators (e.g., prostaglandin) contributing to pain and sensitization [21,24]. The most widely studied cytokines in joint pain include IL-1β, TNFα, IL-6 and IL-17. A single injection of TNFα into regular rat knee joints produced persistent sensitization of nociceptive Aδ- and C-fibers leading to mechanical allodynia and hyperalgesia. Pharmacological neutralization of TNFα in turn prevented cytokine-induced sensitization [9]. Similar results were observed for IL-6 injections although sensitization was limited to C-fibers [10]. Significant correlations between both TNFα and IL-6 and pain and function have also been reported for human OA [17,18]. Our results indicate weak to moderate correlations between SF IL-6 levels and OA severity as well as knee function. Higher synovial IL-6 concentrations were associated with increased K&L scores which is in line with recent findings [25]. A prospective, population-based study including healthy, middle-aged women found increasing serum levels of IL-6 to be independent predictors of the appearance of radiographic knee OA [26]. Previously, it was reported that SF IL-6 is associated with synovitis in the parapatellar subregion and knee pain [17]. In our study population, increasing SF IL-6 levels correlated with poorer knee function (OKS-12) but not pain intensity alone (NRS). Increasing stiffness with higher IL-6 levels has also been described for serum samples of knee OA patients [27].

In contrast to previous findings [18,19], we couldn’t demonstrate associations between TNFα levels in SF and clinical parameters (K&L, NRS, OKS-12). TNFα inhibition using monoclonal antibodies has been effective for treating both rheumatoid and psoriatic arthritis. In OA, TNFα inhibition showed mixed results. Although there might be a potential positive effect on the progression of erosive hand OA [28], several studies have failed to demonstrate pain amelioration using TNFα inhibitors [29]. Compared to RA, OA presents with a lower level of joint inflammation. Thus, targeting TNFα pathways to alleviate pain might not be appropriate in joints with low-grade inflammation. On the other hand, significant associations between synovial TNFα levels and both pain on movement and at rest have been reported in symptomatic knee OA patients [19]. The contradicting findings might be the result of adjustment for potentially influencing factors, a greater number of study patients or the use of different pain questionnaires [19]. Interestingly, IL-1 and IL-17 concentrations were below the detection level although IL-1 is the main macrophage and IL-17 the main Th17 secreted cytokine. It has been suggested that these cytokines could play a more relevant role in early than late OA inducing the expression of downstream mediators including CXCL1 and IL-7 [5,30,31]. Indeed, SF CXCL1 levels were moderately associated with OA severity. This is in line with in-vitro studies demonstrating the apoptotic effect of CXCL1 on OA chondrocytes [32].

Several studies characterized the inflammatory milieu of SF in knee OA [5,22,33,34,35]. The majority of inflammatory mediators can be detected in the SF of both healthy and OA patients. Nevertheless, significant differences in concentrations levels were observed [16,33]. Despite different cytokine profiles of OA and normal joints correlation studies between inflammatory mediators and clinical data are rare. Here, we present the results of an extensive analysis correlating a total of 28 inflammatory mediators with OA severity, pain intensity and knee function. We identified 5 mediators of potential clinical relevance due to moderate associations with both knee pain and function. Interestingly, these mediators include anti-inflammatory interleukins (IL-10, IL-13) and growth factors (SCGF-β, VEGF). Although the pathophysiological processes in OA joints are predominately characterized by pro-inflammatory mediators driving catabolic effects, anti-inflammatory cytokines may play a more important role as previously suspected. In OA, the major anti-inflammatory cytokines represent IL-4, IL-10 and IL-13 [22]. IL-10 shows chondroprotective effects by inhibiting MMPs and apoptosis of chondrocytes and reduces the release of pro-inflammatory TNFα and IL-1β [22,36,37,38]. Furthermore, experimental studies using different murine models of pain demonstrated significant involvement of IL-10 in pain processing [39]. Intrathecal administration of an IL4–10 fusion protein inhibited mechanical and thermal hyperalgesia in a mouse model of inflammatory pain [39]. Our results also indicate involvement of anti-inflammatory mediators (IL-10 and IL-13) in human OA pain. Surprisingly, increasing SF levels of both IL-10 and IL-13 were significantly associated with greater pain and poorer knee function. Since IL-10 reduces TNFα and IL-1β expression [22,36,37,38] and intracellular protein levels of IL-10 in chondrocytes increase with cartilage damage [36], we hypothesize that the correlation between anti-inflammatory mediators and pain represents a counteractive response to the detrimental catabolic effects of pro-inflammatory cytokines. To maintain the balance between anti- and pro-inflammatory mediators within this complex cytokine network and to antagonize the pro-inflammatory mechanisms leading to OA progression and pain, anti-inflammatory cytokines might be released. Displaying the competition between pro- and anti-inflammatory processes IL-10 and IL-13 might serve as a marker for OA-related pain, although a causal nociceptive effect seems unlikely. Since IL-10-induced pain amelioration in rodent models of inflammatory pain was mediated through inhibition of glial activation in the spinal cord and decreased cytokine levels in dorsal root ganglia [39], analgesic effects of intraarticular anti-inflammatory mediators, including IL-10, might be weaker and need further investigation.

Moreover, our results highlight the role of growth factors in OA-related pain. Both SCGF-β and VEGF levels correlated significantly with NRS and OKS-12 scores. VEGF acts as survival factor for growth plate chondrocytes during development and is crucial for enchondral ossification [40,41,42]. Nevertheless, recent findings from a meta-analysis of genome-wide association studies identified significant associations between the VEGF gene and OA [43]. Accordingly, several experimental and translational studies support interactions between increased VEGF levels and OA progression [44]. Intraarticular VEGF injection in knee joints of healthy mice led to synovial hyperplasia, subchondral bone sclerosis and cartilage degradation [45]. In line with these findings, our results demonstrate that K&L scores significantly correlate with VEGF levels. In addition, VEGF signaling seems to be involved in OA-related pain [44]. Mechanistically, VEGF-induced angiogenesis promotes inflammation and, indirectly, sensory neuron ingrowth into the osteochondral junction, which in turn leads to sensitization of primary nociceptive afferents. Furthermore, VEGF might directly activate sensory neurons [44]. Indeed, in several rodent models of pain, including cancer pain, it was demonstrated that VEGF signaling plays a significant role in the pathophysiology of pain [46]. In line with these findings, we report significant associations between OA-related pain and VEGF levels. Thus, targeting VEGF signaling might be a promising pharmacological approach to treat pain in patients suffering from OA [15], although further investigations are needed.

Interestingly, we also found significant correlation between SCGF-β and OA severity, pain and knee function. To date, little has been known about this secreted sulfated glycoprotein. SCGF is encoded by the CLEC11A gene and belongs to the C-type lectin superfamily. It stimulates primitive hematopoietic progenitor cells [47,48] and seems to play a role in different types of malignant diseases, including breast and lung cancer, as well as hepatocellular carcinoma [49,50]. To our knowledge, limited data are available regarding the pathophysiological role of SCGF. In situ hybridization of whole mouse fetuses revealed SCGF mRNA expression around skeletal tissue including proliferating chondrocytes and periosteum with little expression levels in resting or hypertrophic chondrocytes. Yet, these findings refer to the full size SCGF form SCGF-α (35 kDa). In contrast, SCGF-β is shorter (27 kDa) and characterized by a deletion within a conserved carbohydrate recognition domain [47,48]. Whether there is a role of SCGF in OA needs to be investigated. Our results suggest significant correlations with OA progression and pain. Due to expression in active osteochondral tissue SCGF indeed might be a new player in the cytokine network underlying the pathophysiology of OA.

However, the study was limited by the fact that no control group was included. Thus, a comparison of cytokine/pain levels between healthy patients without OA and our study population was not possible. Furthermore, cytokine levels might be influenced, inter alia, by age, gender, BMI, disease stage and joint inflammation. Adjustment to possible confounding factors was not performed in our analysis. Although OA severity ranged from K&L scores 2 to 4, all patients suffered from clinically relevant advanced OA and therefore underwent surgery. Thus, the reported cytokine profile represents the inflammatory status of patients requiring surgery due to OA-induced symptoms and/or functional limitations. Since cytokine analyses were performed based on SF samples from a one-time aspiration the trajectory of these cytokines prior to surgery was not monitored. Cytokine variability across patients was not tested in detail but might affect the interpretation of the identified inflammatory mediators. Generalization of our findings has to be performed with caution, since early and intermediate OA stages might present a different cytokine pattern. Explorative analysis of the < 25th and > 75th percentile of UC and BC patients for IL-10, IL-12, IL-13, SCGFβ and VEGF revealed that the inflammatory profile was relatively consistent across patients within each group. For all cytokines, the same patients (2 for UC, 4 for BC) showed concentrations levels > 75th percentile. Similar results were obtained for patients belonging to the lower quartile (< 25th percentile), although variability was higher.

Further randomized clinical trials, including age-, gender- and BMI-matched patients are needed to conclusively untangle the role of the cytokine network in OA-related pain. For this future work our results provide a detailed map of the inflammatory pattern in OA-related pain.

## Figures and Tables

**Table 1:** Study population.

	Total Study Population	UC OA	BC OA
Number of patients, *n*	34	14	20
Gender, *n* (%)			
Male	14 (41.2%)	9 (64.3%)	5 (25.0%)
Female	20 (58.8%)	5 (35.7%)	15 (75.0%)
Age, years	67.38 ± 10.48 (40–89)	66.71 ± 10.23 (50–89)	67.85 ± 10.89 (40–83)
BMI (kg/m²)	30.74 ± 5.78 (21.00–43.00)	29.43 ± 5.36 (22.00–40.00)	31.65 ± 6.02 (21.00–43.00)
K&L score, *n* (%)			
2	5 (15.1%)	2 (14.3%)	3 (15.8%)
3	13 (39.4%)	9 (64.3%)	4 (21.0%)
4	15 (45.5%)	3 (21.4%)	12 (63.2%)
Knee pain, NRS (0–10)	7.12 ± 2.29 (2.00–10.00)	6.21 ± 2.39 (2.00–10.00)	7.75 ± 2.05 (3.00–10.00)
OKS-12 (Pt. 12–60)	35.15 ± 7.68 (19.00–46.00)	32.21 ± 6.69 (19.00–44.00)	37.32 ± 7.80 (22.00–46.00)

Demographic and clinical parameters of the study population are presented. Data are presented as mean ± standard deviation (SD; range). OA = osteoarthritis; UC = unicompartmental; BC = bicompartmental; BMI = body mass index; NRS = numerical rating scale; OKS-12 = Oxford Knee Score; K&L score = Kellgren and Lawrence score.

**Table 2:** Correlation analyses between inflammatory mediators in SF and clinical parameters.

Mediator	Concentration in SF [pg/mL]	K&L Score		Knee Pain, NRS (0–10)		OKS, (Pt. 12–60)	
	Mean ± SD (Range)	r	*p*	r	*p*	r	*p*
IL-6	277.4 ± 368.7 (7.2–1666.4)	0.37	0.035 *	0.23	0.18	0.41	0.017 *
IL-7	35.3 ± 13.1 (14.5–69.7)	0.26	0.14	0.39	0.023 *	0.21	0.23
IL-8	405.1 ± 694.9 (13.2–3419.8)	0.47	0.006 **	−0.04	0.81	0.10	0.59
IL-10	35.4 ± 16.5 (13.7–88.3)	0.22	0.21	0.47	0.005 **	0.35	0.047 *
IL-12	284.1 ± 150.5 (123.8–786.8)	0.22	0.22	0.47	0.005 **	0.36	0.037 *
IL-13	38.2 ± 16.6 (16.5–97.1)	0.20	0.28	0.47	0.005 **	0.43	0.012 *
IL-15	9.1 ± 4.2 (2.8–22.7)	0.14	0.45	−0.20	0.26	0.17	0.36
IL-16	1122.4 ± 757.4 (378.2–4256.3)	0.28	0.11	−0.16	0.38	0.29	0.10
IL-18	100.8 ± 53.6 (43–321.6)	0.14	0.45	−0.10	0.58	0.36	0.043 *
βNGF	9.6 ± 2.9 (5.4–20.3)	0.10	0.60	0.05	0.79	0.40	0.021 *
IFNγ	142.7 ± 73.3 (45.5–406.9)	0.35	0.049 *	0.35	0.044 *	0.34	0.05
IFNα2	126.4 ± 21.40 (80.92–175.7)	0.20	0.26	−0.12	0.49	0.27	0.13
LIF	47.2 ± 25.2 (10.1–108.6)	0.25	0.16	−0.29	0.10	0.22	0.22
M-CSF	85.80 ± 51.90 (28.42–283.8)	0.22	0.23	−0.08	0.63	0.20	0.27
MIP-1β	40 ± 24.1 (12.8–133.1)	−0.06	0.76	−0.10	0.59	0.07	0.70
CCL2	114.6 ± 293.2 (8.6–1655.8)	0.02	0.93	0.14	0.43	0.24	0.18
CCL5	115.8 ± 329.5 (0.1–1791.1)	0.12	0.49	−0.01	0.97	0.34	0.05
CCL7	82.92 ± 26.4 (30.80–141.4)	0.001	0.99	−0.08	0.65	0.18	0.30
CCL27	500.0 ± 155.3 (284.0–1104)	0.03	0.88	−0.12	0.50	0.03	0.86
SCF	346.7 ± 102.9 (157.2–623.2)	0.09	0.61	−0.33	0.06	0.10	0.58
SCGF-β	32337.5 ± 25341.6 (7435–147588)	0.43	0.013 *	0.35	0.040 *	0.53	0.002 **
TNF-a	5 ± 3.1 (0.5–12.2)	0.34	0.053	0.27	0.12	0.32	0.07
VEGF	2180.3 ± 2087.7 (569.4–10159.9)	0.39	0.025 *	0.44	0.009 **	0.37	0.034 *
CXCL1	232.4 ± 316.7 (54.76–1733)	0.49	0.004 **	0.08	0.64	0.30	0.09
CXCL9	1972 ± 1378 (368.5–7445)	0.15	0.42	0.06	0.73	0.38	0.028 *
CXCL12	110.7 ± 31.41 (58.17–208,7)	0.19	0.30	−0.22	0.21	0.02	0.89

The Pro-Human Cytokine Multiplex Assay (Bio-Rad) was used to analyze cytokines in synovial fluid (SF) samples. Cytokine and chemokine concentrations were calculated by reference to the standard curve. The sensitivity of the multiplex kit was < 5 pg/mL. Concentration levels of IL-1α, IL-1β, IL-2, IL-4, IL-5, IL-9 and IL-17 were below the detection level. Synovial concentrations of inflammatory mediators are presented as mean ± standard deviation (range). Correlation analyses between inflammatory mediators in SF and severity of knee osteoarthritis (K&L score), knee pain (NRS) as well as knee function (OKS) were performed using Spearman’s rank correlation coefficient (r). *p*-Values < 0.05 were considered statistically significant and are indicated with asterisks: * *p* < 0.05; ** *p* < 0.01. SF = synovial fluid; CCL = C-C motif ligand; CXCL = C-X-C motif ligand; IFN = interferon; IL = interleukin; LIF = leukaemia inhibitory factor; MCP = monocyte chemotactic protein; MIP = macrophage inflammatory protein; M-CSF = macrophage colony-stimulating factor; NGF = nerve growth factor; SCF = stem cell factor; SCGF = stem cell growth factor; TNF = tumor necrosis factor; VEGF = vascular endothelial growth factor.

**Table 3:** Different cytokine patterns in UC and BC OA.

Mediator	UC OA Median (IQR)	BC OA Median (IQR)	*p*-Value
IL-7	29.04 (23.79, 34.56)	34.64 (29.34, 48.40)	0.0321 *
IL-8	38.96 (27.36, 86.69)	208.2 (44.45, 615.5)	0.0390 *
IL-10	26.29 (21.95, 33.19)	36.21 (26.77, 52.14)	0.0047 **
IL-12	207.8 (160.7, 248.8)	279.5 (213.0, 425.9)	0.0200 *
IL-13	28.20 (22.55, 37.07)	38.54 (29.99, 53.11)	0.0264 *
IFN-γ	112.7 (85.34, 138.6)	138.6 (103.0, 187.0)	0.0439 *
VEGF	1178 (747.1, 1762)	1855 (1358, 3428)	0.0108 *
CXCL1	95.69 (73.09, 134.1)	171.1 (119.0, 258.4)	0.0097 **

Significant differences in concentration levels between UC and BC OA were observed for the presented inflammatory mediators using Mann–Whitney *U* test. Concentration levels are presented as median (IQR) in pg/mL and were calculated by reference to the standard curve. The sensitivity of the multiplex kit was < 5 pg/mL. Significant differences are indicated with asterisks: * *p* < 0.05; ** *p* < 0.01. IL = interleukin; IFN = interferon; VEGF = vascular endothelial growth factor; C-X-C motif ligand 1 = CXCL1, IQR = interquartile range.
